# Articular Manifestations in Inflammatory Bowel Disease: A Retrospective Study From a Moroccan Tertiary Care Center

**DOI:** 10.7759/cureus.112882

**Published:** 2026-07-17

**Authors:** Fatimaezzahra Lairani, Jihane Rizkou, Fatimaezzahra Chakor, Hala Aouroud, Oussama Nacir, Adil Ait Errami, Sofia Oubaha, Zouhour Samlani, Khadija Krati

**Affiliations:** 1 Gastroenterology and Hepatology, Mohammed VI University Hospital Center, Marrakech, MAR

**Keywords:** articular manifestations, crohn disease, inflammatory bowel disease, morocco, sacroiliitis, spondyloarthritis, ulcerative colitis

## Abstract

Background: Articular manifestations are among the most common extraintestinal manifestations of inflammatory bowel disease (IBD) and may precede, accompany, or follow intestinal symptoms. Their recognition is clinically important because delayed diagnosis can lead to functional impairment and structural damage.

Methods: We conducted a retrospective descriptive study in the Department of Hepato-Gastroenterology at Mohammed VI University Hospital, Marrakech, Morocco. Patients with confirmed IBD and associated rheumatologic manifestations who were followed between December 2023 and December 2024 were included. Epidemiological, clinical, biological, radiological, therapeutic, and outcome data were extracted from medical records and analyzed using Microsoft Excel 2019 (Microsoft Corporation, Redmond, USA).

Results: Among 150 patients with IBD, 60 had articular manifestations, corresponding to a prevalence of 40%. Crohn disease (CD) was present in 33 patients (55%), ulcerative colitis (UC) in 24 patients (40%), and indeterminate colitis in three patients (5%). There was a female predominance, with 41 women (68.3%) and 19 men (31.7%). Articular symptoms occurred after the diagnosis of intestinal disease in 37 patients (61.7%), before it in 17 patients (28.3%), and concomitantly in six patients (10%). Mixed axial and peripheral involvement was the most frequent pattern, observed in 35 patients (58.3%), followed by isolated peripheral involvement in 16 patients (26.7%) and isolated axial involvement in nine patients (15%). Inflammatory back pain was reported in 44 patients (73.3%), buttock pain in 37 patients (61.7%), and radiographic sacroiliitis in 25 patients (41.7%). Human leukocyte antigen B27 (HLA-B27) was positive in three of 33 tested patients (9.1%).

Conclusions: Articular manifestations were frequent in this Moroccan IBD cohort and were dominated by mixed axial and peripheral involvement. Systematic screening for musculoskeletal symptoms in patients with IBD and close collaboration between gastroenterologists and rheumatologists are essential for early diagnosis, optimized management, and prevention of functional impairment.

## Introduction

Inflammatory bowel disease (IBD), including Crohn disease (CD), ulcerative colitis (UC), and indeterminate colitis, is a chronic immune-mediated disorder characterized by relapsing intestinal inflammation and a broad spectrum of systemic manifestations. Extraintestinal manifestations can involve the skin, eyes, hepatobiliary tract, and musculoskeletal system, and their presence may influence prognosis, treatment selection, and quality of life [1,2].

Musculoskeletal involvement is considered one of the most frequent extraintestinal manifestations of IBD. It includes peripheral arthritis, inflammatory back pain, sacroiliitis, axial spondyloarthritis, enthesitis, dactylitis, and, less commonly, chronic erosive arthropathy [2-4]. The clinical course is heterogeneous. Type 1 peripheral arthritis is often acute, pauciarticular, and associated with intestinal flares, whereas type 2 peripheral arthritis and axial disease may evolve independently of intestinal disease activity [3,4].

The gut-joint axis is supported by shared genetic susceptibility, altered intestinal permeability, dysbiosis, immune cell trafficking, and cytokine pathways such as tumor necrosis factor alpha and interleukin-23/interleukin-17 signaling [5,6]. These mechanisms explain why therapies used to control intestinal inflammation, particularly anti-tumor necrosis factor agents, may also improve articular disease [2,7].

In daily practice, diagnosis can be challenging because joint symptoms may precede intestinal manifestations, may be attributed to mechanical disorders, or may remain radiologically silent during early disease. Classification criteria for spondyloarthritis and the increasing role of magnetic resonance imaging (MRI) have improved early recognition, particularly in patients with inflammatory back pain and normal radiographs [8,9].

The aim of this study was to describe the prevalence, clinical patterns, biological and radiological profile, and therapeutic management of articular manifestations in patients with IBD followed at a Moroccan tertiary-care center.

## Materials and methods

Study design and setting

This was a retrospective descriptive study conducted in the Department of Hepato-Gastroenterology at Mohammed VI University Hospital, Marrakech, Morocco. The study evaluated patients who were followed between December 2023 and December 2024.

Study population

Patients were eligible if they had a confirmed diagnosis of IBD and at least one associated articular or rheumatologic manifestation. The diagnosis of IBD was based on clinical, biological, endoscopic, radiological, and histological findings. Patients with incomplete medical records or an uncertain diagnosis of IBD were excluded.

Data collection

Data were extracted from hospitalization registers, medical records from the Department of Hepato-Gastroenterology, the computerized hospital system, and available multidisciplinary assessments. A standardized data collection sheet was used to record epidemiological variables, IBD phenotype, timing and type of articular involvement, biological markers, imaging findings, therapeutic strategies, and clinical outcomes.

Definitions

Articular involvement was classified as axial, peripheral, or mixed axial-peripheral disease. Axial disease included inflammatory back pain, buttock pain, radiographic- or MRI-detected sacroiliitis, and spondyloarthritis. Peripheral arthritis was classified as type 1 or type 2 according to the commonly used clinical distinction in IBD-associated arthropathy [3,4].

Statistical analysis

Data were entered and analyzed using Microsoft Excel 2019. Qualitative variables were presented as numbers and percentages. Because of the descriptive retrospective design, no multivariable inferential analysis was performed.

## Results

Prevalence and inflammatory bowel disease phenotype

Among 150 patients with IBD followed during the study period, 60 patients had articular manifestations, corresponding to a prevalence of 40%. CD was the most frequent underlying IBD subtype, observed in 33 of 60 patients (55%), followed by UC in 24 of 60 patients (40%) and indeterminate colitis in three of 60 patients (5%). The distribution of patients according to IBD subtype is shown in Table 1.

**Table 1:** Distribution of patients according to IBD subtype Values are presented as the number of patients and percentage. IBD: inflammatory bowel disease; CD: Crohn disease; UC: ulcerative colitis

IBD subtype	Number of patients	Percentage
CD	33	55.00%
UC	24	40.00%
Indeterminate colitis	3	5.00%
Total	60	100%

Demographic characteristics

A female predominance was noted, with 41 women (68.3%) and 19 men (31.7%), corresponding to a female-to-male ratio of 2.1. Most patients were young or middle-aged adults: 12 patients (20%) were younger than 25 years, 41 patients (68.3%) were aged 25 to 50 years, and seven patients (11.7%) were older than 50 years. Demographic characteristics of the study population are summarized in Table 2.

**Table 2:** Demographic characteristics of the study population Values are presented as the number of patients and percentage.

Variable	Number of patients	Percentage
Female sex	41	68.30%
Male sex	19	31.70%
Age younger than 25 years	12	20.00%
Age 25-50 years	41	68.30%
Age older than 50 years	7	11.70%

Timing of articular manifestations

Articular manifestations occurred after the diagnosis of IBD in 37 of 60 patients (61.7%), before the digestive diagnosis in 17 of 60 patients (28.3%), and concomitantly with the first intestinal flare in six of 60 patients (10%). Among patients whose articular symptoms preceded IBD diagnosis, nine of 17 patients (52.9%) had CD and eight of 17 patients (47.1%) had UC.

Clinical patterns of joint involvement

Mixed axial and peripheral involvement was the most frequent clinical pattern, affecting 35 of 60 patients (58.3%). Isolated peripheral involvement was observed in 16 of 60 patients (26.7%), whereas isolated axial involvement was reported in nine of 60 patients (15%). This distribution highlights the need for systematic evaluation of both axial and peripheral symptoms during IBD follow-up. Patterns of articular involvement are presented in Table 3.

**Table 3:** Patterns of articular involvement Values are presented as the number of patients and percentage.

Pattern	Number of patients	Percentage
Mixed axial and peripheral involvement	35	58.30%
Isolated peripheral involvement	16	26.70%
Isolated axial involvement	9	15.00%

Axial manifestations

Inflammatory back pain was reported in 44 of 60 patients (73.3%). Pain was predominantly lumbar in 43 of 60 patients (71.7%), while dorsal pain was reported in one of 60 patients (1.7%) and cervical pain in 19 of 60 patients (31.7%). Buttock pain was present in 37 of 60 patients (61.7%), including bilateral buttock pain in 22 of 60 patients (36.7%), unilateral buttock pain in four of 60 patients (6.7%), and alternating buttock pain in 11 of 60 patients (18.3%).

Peripheral manifestations

Type 1 peripheral arthritis was observed in 12 of 60 patients (20%), including seven patients with CD and five patients with UC. Type 2 peripheral arthritis was found in four of 60 patients (6.7%), including two patients with CD and two patients with UC. Peripheral involvement was mainly asymmetric and most frequently affected the knees, ankles, and shoulders.

Biological findings

Biological assessment frequently demonstrated systemic inflammation. Leukocytosis greater than 10000 cells/mm³ was present in nine of 60 patients (15%), hypochromic microcytic anemia in 18 of 60 patients (30%), elevated erythrocyte sedimentation rate in 41 of 60 patients (68.3%), and elevated C-reactive protein in 32 of 60 patients (53.3%). Fecal calprotectin was performed in 22 patients and was elevated in 13 of 22 patients (59.1%). Human leukocyte antigen B27 (HLA-B27) testing was performed in 33 patients and was positive in three of 33 patients (9.1%). Rheumatoid factor was tested in 17 patients and was positive in two of 17 patients (11.8%). Joint aspiration was performed in four patients with arthritis and showed inflammatory synovial fluid. The main biological and radiological findings are summarized in Table 4.

**Table 4:** Main biological and radiological findings Percentages were calculated according to the number of patients tested or assessed for each finding. HLA-B27: human leukocyte antigen B27

Finding	Number tested or assessed	Positive or abnormal results, n (%)
Leukocytosis greater than 10000 cells/mm³	60	9 (15.0%)
Hypochromic microcytic anemia	60	18 (30.0%)
Elevated erythrocyte sedimentation rate	60	41 (68.3%)
Elevated C-reactive protein	60	32 (53.3%)
Elevated fecal calprotectin	22	13 (59.1%)
Positive HLA-B27	33	3 (9.1%)
Radiographic sacroiliitis	60	25 (41.7%)
Coxitis	60	8 (13.3%)

Radiological findings

Pelvic radiography was performed in all 60 patients. Sacroiliitis was identified in 25 of 60 patients (41.7%). It was bilateral in 17 of 25 patients (68%) and unilateral in eight of 25 patients (32%). Radiographic staging showed stage 1 sacroiliitis in two of 25 patients (8%), stage 2 in six of 25 patients (24%), stage 3 in 13 of 25 patients (52%), and stage 4 in four of 25 patients (16%). Coxitis was observed in eight of 60 patients (13.3%), being bilateral in five of eight patients (62.5%) and unilateral in three of eight patients (37.5%). Spinal radiographs showed abnormalities such as vertebral squaring, Romanus lesions, syndesmophytes, and ankylosis in selected patients. MRI contributed to the identification of active inflammatory lesions when early axial spondyloarthritis was suspected [8,9].

Relationship with intestinal disease activity

The relationship between intestinal disease activity and joint manifestations varied according to the type of articular involvement. Type 1 peripheral arthritis was most closely associated with intestinal flares, whereas type 2 peripheral arthritis and axial manifestations were less consistently correlated with intestinal activity. This observation is consistent with the recognized clinical behavior of IBD-associated arthropathy [3,4].

Therapeutic management

Management was individualized according to IBD activity, type of articular involvement, severity, and the presence of intestinal or rheumatologic complications. Treatment strategies included optimization of IBD therapy, analgesics, cautious short-term use of nonsteroidal anti-inflammatory drugs when appropriate, corticosteroids, immunomodulators, biologic therapy, and surgical management for selected intestinal complications. Multidisciplinary collaboration between gastroenterologists, rheumatologists, radiologists, and surgeons was central to decision-making.

## Discussion

This retrospective study found articular manifestations in 60 of 150 patients with IBD, corresponding to a prevalence of 40%. This places our cohort in the upper range of prevalences reported in international studies. The ECCO consensus estimates that extraintestinal manifestations, particularly musculoskeletal manifestations, are common in IBD and may require dedicated multidisciplinary assessment [2]. Large cohort studies have similarly emphasized that musculoskeletal symptoms represent a major clinical burden in IBD [1,10].

CD was more frequent than UC among patients with articular involvement in our series, being observed in 33 of 60 patients (55%), compared with UC in 24 of 60 patients (40%). This finding is consistent with several international studies suggesting that musculoskeletal manifestations are slightly more common in CD than in UC [1,10,11]. Possible explanations include the transmural inflammatory pattern of CD, differences in systemic inflammatory load, and distinct immunogenetic susceptibility.

The female predominance observed in our study, with 41 women (68.3%) and 19 men (31.7%), differs from some historical descriptions but is consistent with more recent real-life cohorts in which women frequently report extraintestinal and rheumatologic symptoms. This may reflect true sex-related susceptibility, differences in health-seeking behavior, or referral patterns. Because our design was retrospective and single-center, this result should be interpreted cautiously.

A clinically important finding was that articular symptoms preceded the diagnosis of IBD in 17 of 60 patients (28.3%). This supports the need to search for digestive symptoms, anemia, inflammatory markers, and fecal calprotectin abnormalities in patients presenting with unexplained spondyloarthritis or recurrent peripheral arthritis. Conversely, patients with IBD should be asked systematically about inflammatory back pain, buttock pain, enthesitis, and peripheral joint swelling [2,8].

Mixed axial and peripheral involvement was the dominant pattern in our cohort, affecting 35 of 60 patients (58.3%). This is relevant because mixed forms may be more disabling and may require therapeutic strategies that control both intestinal and rheumatologic inflammation. Peripheral type 1 arthritis often parallels digestive disease activity, while type 2 peripheral arthritis and axial disease may persist independently of intestinal control [3,4]. This distinction should guide follow-up and treatment escalation.

Radiographic sacroiliitis was found in 25 of 60 patients (41.7%). However, radiographs may miss early inflammatory lesions, and MRI has become central to detecting active sacroiliitis, especially when inflammatory back pain is present and plain radiographs are normal [8,9]. In settings where MRI access is limited, clinical screening remains crucial to identify patients who should be referred for rheumatologic evaluation.

HLA-B27 positivity was uncommon in our tested patients, observed in three of 33 patients (9.1%). This is compatible with the concept that IBD-associated spondyloarthritis can occur independently of HLA-B27, particularly in peripheral forms and in some populations with different genetic backgrounds [2,12]. Therefore, a negative HLA-B27 test should not exclude the diagnosis when clinical and imaging features are suggestive.

Treatment of IBD-associated articular manifestations requires careful balancing of digestive and joint disease activity. Nonsteroidal anti-inflammatory drugs can improve axial symptoms but may worsen intestinal disease in some patients and should be used cautiously. Corticosteroids may help peripheral symptoms but are not appropriate for long-term maintenance. Anti-tumor necrosis factor agents are effective for both intestinal inflammation and spondyloarthritis, whereas other targeted therapies should be selected according to the dominant phenotype and current guideline recommendations [2,7,13].

Overall, our findings are consistent with broader reviews describing the clinical spectrum of extraintestinal manifestations in IBD, systematic estimates of axial and peripheral spondyloarthritis prevalence in IBD populations, and international recommendations emphasizing individualized management of axial spondyloarthritis [14-16]. These data support the need for early recognition of musculoskeletal symptoms and coordinated management between gastroenterologists and rheumatologists.

The main limitations of this study are its retrospective design, single-center setting, and possible missing data. MRI, HLA-B27 testing, and rheumatologic assessment were not uniformly performed in all patients. In addition, the descriptive design does not allow identification of independent predictors of joint involvement. Despite these limitations, the study provides useful real-life data from a Moroccan tertiary-care center and supports systematic screening for musculoskeletal manifestations in IBD.

## Conclusions

Articular manifestations were frequent in this Moroccan tertiary-care cohort, affecting 60 of 150 patients with inflammatory bowel disease (40%). They were predominantly characterized by mixed axial and peripheral involvement, observed in 35 of 60 patients (58.3%), followed by isolated peripheral involvement in 16 patients (26.7%) and isolated axial involvement in nine patients (15%). Articular symptoms preceded the diagnosis of inflammatory bowel disease in 17 of 60 patients (28.3%), highlighting the importance of considering underlying inflammatory bowel disease in patients with unexplained inflammatory rheumatologic symptoms.

Inflammatory back pain, buttock pain, sacroiliitis, and peripheral arthritis should be actively screened for during inflammatory bowel disease follow-up. Early collaboration between gastroenterologists and rheumatologists, appropriate imaging, and individualized treatment are essential to improve functional outcomes and prevent structural complications.
